# Presentation of a participatory approach to develop preventive measures to reduce COVID-19 transmission in child care

**DOI:** 10.1186/s12995-021-00316-0

**Published:** 2021-07-14

**Authors:** Mathias Diebig, Susan Gritzka, Nico Dragano, Peter Angerer

**Affiliations:** 1 Institute of Occupational, Social and Environmental Medicine, Heinrich Heine University Düsseldorf, Universitätsstr. 1, 40225 Düsseldorf, Germany; 2 Institute of Medical Sociology, Heinrich Heine University Düsseldorf, Düsseldorf, Germany

**Keywords:** Preventive measures, COVID-19 transmission, Participation, Work design

## Abstract

**Background:**

It can be suspected that work in child care facilities is associated with an elevated exposure risk towards SARS-CoV-2 infections. It is still unclear under which conditions employees in those facilities can safely pursue their work. Preventive workplace-related measures to reduce transmission dynamics in this work environment need to be developed. These measures need to build on a solid scientific foundation and be ready for practical use at the same time. Therefore, the aim of the study is to present a participatory approach to identify, minimize, and eliminate workplace-specific COVID-19 transmission within child care. The approach presented combines quantitative as well as qualitative elements and includes a screening of critical workplace conditions and the development of preventive measures to foster a safe workplace design.

**Methods:**

First, 428 employees of different child care facilities in a large German city reported their subjective risk of infection, fear of infection, and support received by the employer. Second, the participants commented in detail about high risk conditions during work. Third, employees provided suggestions for preventive measures. We conducted a qualitative analysis of free text answers to evaluate which aspects are perceived as critical from an employee perspective.

**Results:**

Participants provided valuable and practicable ideas on how to design and improve preventive measures to reduce COVID-19 transmission in child care dealing with structural conditions, the interaction with the parents, the implementation of preventive measures and recommendations for policy makers.

**Conclusions:**

These new insights help to organize pandemic risk management in order to align theoretical based measures with the practical realization. We encourage researchers to adapt the approach presented to other work areas in order to foster participation of employees in work design to reduce COVID-19 transmission.

**Supplementary Information:**

The online version contains supplementary material available at 10.1186/s12995-021-00316-0.

## Introduction

The coronavirus disease 2019 (COVID-19) caused by SARS-CoV-2 has become one major global challenge for modern society. Public health interventions targeting the slowdown of transmission dynamics have a strong impact on society, policy, and economy. After acute public health interventions (e.g., lockdowns) have been implemented in the beginning of the pandemic, calls for targeted interventions to reboot the working life are growing [1]. Questions regarding how and under which conditions individuals can safely return to their workplaces become increasingly important. In particular, individuals at workplaces associated with a high infection risk demand preventive measures combining scientific rigor and practical feasibility. One of these high risk work areas is child care, although the extend of child-to-adult transmission is not yet quantified [2–4]. Child care staff working with young children have a workplace which precludes general protective measures such as social distancing as well as personal protective equipment [5]. Thus, it is crucial to provide them with a series of preventive measures that fit the requirements of early childhood education. We aim to present a participatory approach to identify, minimize or even eliminate workplace-specific infection risks. We focus on workplace stressors arising from the current COVID-19 situation and examine preventive measures to reduce the psychological burden.

Our research design combines quantitative as well as qualitative methods to screen critical work characteristics in a three-step process: First, we collected information on three aspects of workplace conditions (risk of infection, fear of infection and support of employer) using a standardized quantitative questionnaire. Second, we explored causes of these critical aspects to identify why some of them are perceived as problematic using qualitative methods. This mixed methods screening approach is complemented by a third qualitative step in which employees made suggestions on preventive measures. Our participatory process may be used to design a safer workplace during the COVID-19 pandemic – as well as future pandemics – allowing the consideration of workers’ personal view.

## Background

In Germany, children have the possibility to attend kindergarten and day care facilities until school enrollment (on an average 5 years). Employers of these facilities are accountable for occupational health and workplace safety activities. They have to create a work environment that is secure and safe. During the first wave of the pandemic in early 2020, employers received instructions and guidelines on how to re-design child care from their corresponding Federal State Ministry for Family Affairs. This information, however, was only partly evidence-based and implementation was difficult because of the general nature of advises for safety measures. Our approach is located in this information process and displays perceptions as well as recommendations given by the employees of child care facilities, who had to put these preventive measures in practice and work under adjusted working conditions.

This study highlights three aspects that are proposed to be of major importance for the management of COVID-19 at work: risk of infection, fear of infection and support of employer. SARS-CoV-2 spreads person-to-person while the dominant route of transmission is assumed to be respiratory. SARS-CoV-2 has been found in exhaled breath and aerosols [6]. Most transmission occurs through close range contact [7–9]. Research suggests that beside washing or sanitizing hands and avoiding touching one’s face, physical distancing and wearing face masks protects individuals against an infection by SARS-CoV-2 as well [10, 11]. These effective measures to reduce transmission of SARS-CoV-2 can only be partially implemented in child care as they contradict pedagogical concepts. Face covering interferes with core pillars of child education and development, since learning to recognize emotions from facial cues plays a vital role in early childhood, especially for children under 3 years [12, 13]. Facial emotional expressions and their recognition are fundamental to human social interaction because they assumably enable an appropriate prediction of another individual’s emotional state to anticipate future actions [14]. Similarly, maintaining physical distance is particularly difficult as close body contact is sometimes needed with infants in child care settings in order to calm and comfort them or to provide personal hygiene. To conclude, effective prevention cannot easily be applied within child care to reduce the risk of infection for children and employees.

Additionally, we also focus on perceived fear of infection, which may amplify if individuals have risk factors associated with the development of a severe course of disease [7]. Thus, individuals may experience fears about their own health but also about infecting others such as close family members [15]. In addition, the pandemic situation is associated with generalized feelings of insecurity and psychological stress. Due to fear of infection and increased perceived stress employees might stay absent from work in order to avoid risk-prone situations [16]. Recent research also showed that fear of infection critically impairs important work and health outcomes of individuals [17].

Also, the employers’ support is a key topic in this study since it summarizes major considerations for protecting employees. It has been shown that the enactment of policies, practices, and procedures to design safe working conditions by the employer has an effect on employees’ work environment, psychosocial and physical health problems [18, 19]. Employers’ support describes the evaluation of how employees perceive and evaluate the preventive measures implemented by their employers. Results from this research may be used to adjust existing preventive measures by considering the experience of individuals who work in the child care setting and are therefore experts of their own work.

## Methods

### Sample and procedure

We contacted 371 child care facilities and 174 day care institutions in a large city in Western Germany. Participation was voluntary and informed consent was given by all participants. In total, 428 employees from different child and day care facilities participated in an online survey. The mean age of participants was 43.92 years (*SD* = 11.81) ranging from 21 to 70. The majority of participants were female (95%). Most participants worked in child care facilities (79%) most of which were owned by a municipality (44%), a church (16%), a welfare organization (15%), a private sponsor (5%) or other sponsors (20%). The study was approved by the ethics committee at the Medical Faculty of Heinrich Heine University Düsseldorf (no. 2020–1067).

### Instrument

Participants responded to an online-survey and rated their subjective risk of infection, fear of infection and support by the employer. They also stated demographic characteristics. Each topic was explored by three questions: (1) a screening question, (2) a problem question, and (3) a solution question [20, 21]. Screening questions asked whether a certain topic was perceived as critical on a five-point Likert scale with a response format of 1 (*I strongly disagree*) to 5 (*I strongly agree*). Items were adapted from an existing instrument to measure psychosocial work stressors ([22, 23]; risk of infection: “*At my current workplace, there is a risk of being infected with the coronavirus (COVID-19)*”; fear of infection: “*I am afraid of an infection with the coronavirus (COVID-19)*”; support of employer: “*My employer acted quickly to prevent health risks from employees during the coronavirus pandemic*”). The subsequent problem questions identified the root cause of the critical aspects of the different topics. Problem questions presented four to five pre-defined causes of critical workplace conditions. Multiple selection was possible. Pre-defined causes were developed under discussion with ten experts in the field of occupational health. Participants could additionally give a free text answer, which enabled the possibility of a detailed description of the identified problem. Finally, a solution question was presented so that participants could suggest preventive measures to minimize critical workplace conditions. The instrument is displayed in the Additional file 1.

Screening questions were quantitative in nature and analyzed with IBM SPSS 27. Problem questions were on the one hand quantitative (pre-defined causes) and on the other hand qualitative (free text answers). Therefore, a content analysis using MAXQDA 2020 [24] was conducted to identify main topics of critical workplace conditions. We used overarching codes to capture the main issues raised by participants. The material was initially coded by the first author and then discussed by all authors to reduce potential redundancies and ensure comprehensibility as well as replicability. The same procedure was administered for the qualitative solution question.

## Results

### Screening questions

Infection risk (*M* = 4.31, *SD* = 0.98) was perceived as particularly high. The mean value of fear of infection (*M* = 3.10, *SD* = 1.30) was also high, while employers’ support had a medium level (*M* = 2.37, *SD* = 1.16).

### Problem questions

Problem questions were introduced to specify critical working conditions in greater detail and summarize answers on pre-defined causes as well as free text answers. *Answers on pre-defined causes*. Child care staff’s answers on pre-defined causes of problem questions are presented in Table 1. Most participants regarded difficulties with physical distance as the major cause for a high risk of infection (83%). Also, the compliance of other people with safety regulations (30%) and the lack of access to personal protective equipment (10%) were seen as critical aspects regarding infection risk. For fear of infection, participants reported to be afraid of infecting others (37%), infecting themselves (36%) or having a serious course of illness (35%). Participants also reported that they were afraid of the subsequent consequences (e.g., quarantine, economic consequences) of an infection (30%). Employers’ reaction to the pandemic was critically perceived by the absence of materials for personal protection (11%) or the non-adjustment of work processes (9%).

**Table 1:** Child care staff’s answers on pre-defined causes of problem questions

Topic	Answers on problem questions	Percentage
***Risk of infection***	At my current workplace, physical distance to other people (at least 1.5 m) cannot be maintained.	83%
	At work, other people I have to deal with do not comply with the safety regulations.	30%
	I have no access to professional personal protective equipment.	10%
	I feel poorly informed about how to protect myself from an infection in the workplace.	3%
***Fear of infection***	I am afraid of infecting others with the coronavirus.	37%
	I am afraid of getting infected with the coronavirus at work.	36%
	I am afraid of a serious course of illness.	35%
	I am afraid of the possible consequences of an infection (e.g. quarantine, economic consequences).	30%
	I belong to a risk group (e.g. due to a previous illness, age over 60).	16%
***Support of employer***	At my current workplace, not all necessary materials for personal protection against the coronavirus are sufficiently available for me.	11%
	The work processes were not adapted to the current safety regulations.	9%
	No or too little extraordinary information was provided to the child care staff.	4%
	Working from home was not made possible for the child care staff to reduce contact.	1%

Note. Participants could choose multiple pre-defined answers on problem questions

### Free text answers

Participants were also encouraged to give a free text answer on problem questions to provide a detailed description of the critical workplace situation. Regarding the risk of infection, participants described in more detail that the recommended minimum of 1.5-m distance in child care, especially between children and colleagues, could not be maintained in a real-life work situation. Similarly, face masks are considered to be a hindrance. It was also stated that some parents do not fully comply with protective measures, both in private life and in everyday care (including the wearing of face masks, minimum distance or contact reduction). Contact reduction refers to the aspect that children are strictly separated within different groups of one child care facility but then mixed during leisure time when parents meet each other in their free time. Participants assumed an increased risk of infection particularly for children whose parents have high-risk professions for example in the field of medical care. With regard to employees’ fear of infection, respondents were particularly concerned about infecting relatives belonging to a risk group. In addition, some respondents assigned themselves to a risk group (due to age or previous illnesses) and were therefore anxious. Concerning employers’ reaction to the pandemic, the following aspects were of primary importance: In some cases, mainly in the beginning of the pandemic, there was no adequate provision of personal protective equipment. Likewise, in certain circumstances no additional financial resources were received in order to successfully implement protective measures proposed in the facilities. In addition, particular local conditions of the facilities (e.g. small outdoor area) were not sufficiently considered when specifying the protective measures proposed. Information on the protective measures was described as partly inconsistent and not geared to the target group. Child care facilities received lengthy and detailed descriptions of the measures so that they had to study many pages with instructions. Above all, the facilities lacked targeted personnel support in implementing the protective measures, and employees wished to receive support of an expert who visits facilities on site to inspect the specific conditions of each facility.

### Solution question

In a third step, participants were asked to provide suggestions for dealing with the pandemic situation by answering an open question. In total, 123 participants provided a solution to prevent transmission dynamics in their facility. Table 2 summarizes their main proposed solutions arranged around four themes. The first one deals with enhancing the structural organization of child care. This theme subsumes aspects that require timely or financial resources of the employer or policy maker. The second topic concerns interaction between parents and employees. Proposed measures subsume mainly behavioral aspects like informing parents about the pandemic situation to structure communication and encourage sensitivity for the current situation in child care. The third theme deals with the implementation of preventive measures. Communication between policy makers, health experts, and employees of child care facilities is described. Preventive measures in this topic area are mainly based on communication, require minor resources, and ought to render communication processes between the stakeholders more transparent and participative. The fourth issue summarizes measures that aim at support on the political level and mainly contains communication activities. It is described that the appreciation of child care work during the pandemic would help employees deal with the uncertain situation. In sum, the common ground of all measures is a transparent, participative and appreciative communication and interaction between stakeholders within child care to enhance working conditions in the COVID-19 situation.

**Table 2:** Child care staff’s recommendation on preventive measures to minimize COVID-19 transmission in the workplace

Preventive measures on different levels	
***Structural conditions of child care***	
▪ Increase of number of child care staff in kindergartens (e.g. increased staff-children ratio)	
▪ Reduction of the duration of child care (e.g. reduction of hours) during the pandemic	
▪ Reduction of group sizes (children) during the pandemic	
▪ Paid absence of high-risk group	
***Interaction with parents***	
▪ Provision of multilingual and easily comprehensible information material	
▪ Maintenance of the restriction on entering the facility	
▪ Increased control of private behavior of parents as well as the children (reduction of private meetings, which make separated group settings superfluous)	
▪ Review the need for child care if parents have the possibility to stay at home	
***Implementation of preventive measures***	
▪ Provide sufficient protective equipment promptly	
▪ Highlight the sense of purpose of individual protective measures	
▪ Reduction of individual decision-making in the implementation of protective measures	
▪ Better coordination of time scheduling for the implementation of measures	
▪ Participatory involvement of representatives of care institutions in the development of protective measures	
▪ Consideration of the spatial conditions of the facilities when planning mitigating measures	
▪ Targeted control of the facilities with regard to compliance with novel hygiene standards and preventive measures	
***Requested support at the political level***	
▪ Strengthening the public perception and appreciation of the shift in behavior	
▪ Transparent provision of information about the possible occurrence of infections in the facilities	
▪ Introduction of compulsory testing for personnel and children in the facilities (during working hours)	

Note. Questions were originally presented in German. The table presents a translation of the original text

## Discussion

In this study we applied an online participatory approach to identify critical workplace-specific risks associated with COVID-19 transmission within child care and related employee suggestions for preventive measures to reduce risk factors. Although current research shows that infected children predominantly have a mild course [25, 26], viral load and infectivity are comparable to adults [27]. Thus, it is pivotal to implement measures to prevent COVID-19 transmission dynamics within child care. We focused on three topics to organize information collection within the approach presented. Employees of child care facilities informed us about the risk of infection, the fear of infection, and the support of their employer. Furthermore, participants specified critical aspects of these three topics by describing the root causes of the risk factors. This phase of problem identification was complemented by a solution phase in which participants made suggestions for preventive measures to reduce transmission dynamics within child care. These proposed preventive measures can be categorized into four groups. The suggestions refer to different levels: structural conditions, interaction with and behavior of the parents, the process of implementation of measures and the support at the political level. Ultimately, the respondents made tangible recommendations for the adjustment of protective measures.

We assume that the participative approach helps to integrate opinions and expertise of employees within the process of work design during a pandemic. Participation within work design and organizational health is a key aspect for the successful implementation of health-related measures [28]. This approach shows employees that their ideas are taken seriously by the superior level and that they can effectively contribute to the process of creating safe and healthy working conditions [29]. By involving employees, key information on applicability and utility of work design measures can be received. Due to their experience and practical knowledge, child care workers know best about what is feasible and what is not [30]. Further experimental studies have shown that participatory interventions in workplace redesign positively influence the perception of workplace characteristics by employees and thus increase well-being at work [31]. Therefore, we aim to encourage researchers and practitioners to involve employees when discussing the design of work during extraordinary situations like the COVID-19 pandemic.

### Limitations

Although the study has several strengths like the combination of qualitative and quantitative methods of data collection, there are some limitations. First, data was only collected in one city in Germany. Therefore, the generalizability of findings might be limited. Future research should replicate and extend our findings not only within other locations of study but also within other workplaces that are associated with a high infection risk. According to this issue, we were not able to calculate response rates for the sample in this study. We sent the study information to a coordinating office which forwarded the material to the facilities. For this reason, we do not know how many institutions or individuals received the study information. However, recent large-scale studies have revealed that responsiveness seems to be a critical issue within the child care staff population [32].

Second, in the qualitative results we do not differentiate between aspects that were made by a large group of individuals and aspects that only display opinions of a minority of participants. Due to the qualitative nature of our approach, this differentiation was not presented as we mainly focus on collecting information on how to design work more safely. In this context, valuing propositions with regard to frequencies may not be beneficial since the quality of propositions does not depend on the mere quantity of individuals making these propositions. Future research should integrate another subsequent step of evaluation within the proposed participatory approach to include an evaluation of proposed measures by all employees.

Third, we assessed all variables via online self-report questionnaires so that common method bias might have influenced our results. We tested for seriousness of participation at the end of the survey and only included participants who indicated seriousness of their responses [33]. We directly asked participants whether they had answered the questions in a serious manner (“*Please indicate whether you have taken part seriously, so that we can use your answers for our scientific analysis, or whether you were just clicking through to take a look at the survey*”).

## Conclusions

Participants provided valuable ideas about how to design and implement preventive measures to reduce COVID-19 transmission within child care facilities. This approach is promising with regard to generating preventive measures that consider the view of employees who will eventually be confronted with different working conditions. Additionally, we demonstrated that this process may be applied online-based to reduce personal contact. Our approach has yielded some important insights about how to improve work characteristics in a workplace that is associated with a high risk of infection. We encourage researchers to adapt the approach presented to other work areas in order to foster participation in work design of employees to mitigate COVID-19 transmission.

## Supplementary Information

**Additional file 1.**

## Data Availability

The original data are available from the corresponding author on reasonable request.
