# Supplementary material for: Presentation of a participatory approach to develop preventive measures to reduce COVID-19 transmission in child care
Source: J Occup Med Toxicol. 2021 Jul 14;16:26. doi: 10.1186/s12995-021-00316-0 (PMC8278174; doi:10.1186/s12995-021-00316-0)
Supplement: Supplementary file 1 — Additional file 1. [file 12995_2021_316_MOESM1_ESM.docx]

# Appendix: Questionnaire instrument used in the study

| The following section is about the impact of the current coronavirus pandemic (COVID-19) on your work. | | | | | |
| --- | --- | --- | --- | --- | --- |
| At my current workplace, there is a risk of being infected with the coronavirus (COVID-19). | 🞎 I strongly disagree | 🞎 I disagree | 🞎 neutral | 🞎 I agree | 🞎 I strongly agree |
| *Why is there a risk of infection?*   - At my current workplace, physical distance to other people (at least 1.5 meters) cannot be maintained. - At work, other people I have to deal with do not comply with the safety regulations. - I have no access to professional personal protective equipment. - I feel poorly informed about how to protect myself from an infection in the workplace. - Other: | | | | | |
|  | | | | | |
| I am afraid of an infection with the coronavirus (COVID-19). | 🞎 I strongly disagree | 🞎 I disagree | 🞎 neutral | 🞎 I agree | 🞎 I strongly agree |
| *Why are you afraid of an infection with the coronavirus (COVID-19)?*   - I am afraid of infecting others with the coronavirus. - I am afraid of getting infected with the coronavirus at work. - I am afraid of a serious course of illness. - I am afraid of the possible consequences of an infection (e.g. quarantine, economic consequences). - I belong to a risk group (e.g. due to a previous illness, age over 60). - Other: | | | | | |
|  | | | | | |
| My employer acted quickly to prevent health risks from employees during the coronavirus pandemic. | 🞎 I strongly disagree | 🞎 I disagree | 🞎 neutral | 🞎 I agree | 🞎 I strongly agree |
| *Why are you dissatisfied with your employer's actions?*   - At my current workplace, not all necessary materials for personal protection against the coronavirus are sufficiently available for me. - The work processes were not adapted to the current safety regulations. - No or too little information was provided to the child care staff. - Working from home was not made possible for the child care staff to reduce contact. - Other: | | | | | |
|  | | | | | |
| ***In the last section, we asked you about the impact of the coronavirus pandemic (COVID-19) on your work. How do you think the management of the pandemic could be improved?*** | | | | | |

*Note*. Questions were originally presented in German. The table presents a translation of the original text.
